# Conservation of Glomerular Organization in the Main Olfactory Bulb of Anuran Larvae

**DOI:** 10.3389/fnana.2020.00044

**Published:** 2020-07-24

**Authors:** Lukas Weiss, Lucas D. Jungblut, Andrea G. Pozzi, Lauren A. O’Connell, Thomas Hassenklöver, Ivan Manzini

**Affiliations:** ^1^Department of Animal Physiology and Molecular Biomedicine, Justus-Liebig-University Giessen, Giessen, Germany; ^2^Departamento de Biodiversidad y Biología Experimental, IBBEA-CONICET, Universidad de Buenos Aires, Buenos Aires, Argentina; ^3^Department of Biology, Stanford University, Stanford, CA, United States

**Keywords:** amphibians, anura, olfaction, glomeruli, olfactory bulb, evolution

## Abstract

The glomerular array in the olfactory bulb of many vertebrates is segregated into molecularly and anatomically distinct clusters linked to different olfactory functions. In anurans, glomerular clustering is so far only described in *Xenopus laevis*. We traced olfactory projections to the bulb in tadpoles belonging to six distantly related anuran species in four families (Pipidae, Hylidae, Bufonidae, Dendrobatidae) and found that glomerular clustering is remarkably conserved. The general bauplan consists of four unequally sized glomerular clusters with minor inter-species variation. During metamorphosis, the olfactory system undergoes extensive remodeling. Tracings in metamorphotic and juvenile *Dendrobates tinctorius* and *Xenopus tropicalis* suggest a higher degree of variation in the glomerular organization after metamorphosis is complete. Our study highlights, that the anatomical organization of glomeruli in the main olfactory bulb (MOB) is highly conserved, despite an extensive ecomorphological diversification among anuran tadpoles, which suggests underlying developmental constraints.

## Introduction

As in most tetrapods, the olfactory periphery of anuran amphibians is segregated into a main olfactory epithelium (MOE) and a vomeronasal organ (VNO; Eisthen, 1997; Reiss and Eisthen, 2008). Also, several smaller olfactory surfaces have been characterized as specific adaptations to either aquatic olfaction (recessus olfactorius and middle chamber epithelium of the pipid frogs; Helling, 1938; Nowack et al., 2013; Jungblut et al., 2017), aerial olfaction (eminentia olfactoria; Helling, 1938) or possible feeding mechanisms (buccal exposed epithelium; Jungblut et al., 2017). Odorant detection in vertebrates relies on ciliated or microvillous olfactory receptor neurons (ORNs) expressing a single allele belonging to one of several multigene families coding for G-protein coupled olfactory receptors (Buck and Axel, 1991; Dulac and Axel, 1995; Herrada and Dulac, 1997; Liberles and Buck, 2006; Rivière et al., 2009; Greer et al., 2016). In amphibians, each ORN residing in the MOE projects its axon towards one or multiple spheroid neuropil structures (glomeruli) in the main olfactory bulb (MOB; Hassenklöver and Manzini, 2013; Weiss et al., 2020), where synapses with postsynaptic projection neurons are formed.

The glomerular array of many vertebrates is organized in anatomical and functional clusters (Baier and Korsching, 1994; Frontini et al., 2003; Gaudin and Gascuel, 2005; Braubach et al., 2012). A detailed account of glomerular organization in anurans is available only from the fully aquatic *Xenopus laevis* (Manzini and Schild, 2010). *X. laevis* tadpoles have at least two separate odor processing streams from the MOE to spatially segregated glomerular clusters in the MOB. These streams rely on different ORN types, second messenger cascades, and odorant receptor types (Manzini et al., 2002; Gliem et al., 2013). Comparative studies with other anuran species are necessary to understand the relevance of this organization.

The lifecycle of most anurans contains an aquatic larva transforming into an adult frog that dwells on trees, in the water or underground (Duellman and Trueb, 1994; Wells, 2007). However, tadpoles have also diversified and adapted to a variety of aquatic and semiaquatic habitats (Altig and McDiarmid, 1999; Roelants et al., 2011). One distinctive feature among tadpole morphotypes is the oral apparatus and in particular the presence or absence of keratinized mouthparts, which has an impact on the trophic niche occupied by the animals (Orton, 1953; Altig and McDiarmid, 1999). Integrating morphological traits and habitat choice led to the categorization of tadpoles into ecomorphological guilds (Altig and Johnston, 1989). However, little is known about how the sensory system anatomy and function are adapted to the specific demands presented by various habitats.

**Figure 1:**
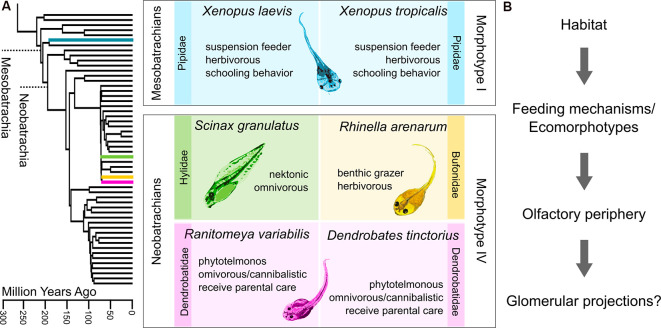
Diversity of anuran tadpoles used in this study. **(A)** The phylogenetic tree on the left is pruned from Pyron (2014), which originally includes 3,309 species. The four families to which the six examined species belong are highlighted. The middle panel describes the six species based on phylogeny, ecology, and morphology. Both *Xenopus* species belong to earlier diverging mesobatrachians, lack keratinized mouthparts (morphotype I), and are thus obligate suspension feeders. The four neobatrachian species can all be classified as morphotype IV. Their developed mouthparts enable them to scrape off food from the substrate. The two dendrobatid species both receive parental care and mostly live in pools in leaf axils or bromeliads. Morphotype distinction follows (Orton, 1953) and ecomorphotypic categorizations are based on (Altig and Johnston, 1989). **(B)** Experimental concept of this study. We tried to examine whether the glomerular organization in the main olfactory bulb (MOB) of tadpoles is influenced by the respective habitat or feeding mechanisms in the distantly related species.

We analyzed and compared the glomerular organization in the MOB of larval anurans belonging to six different species of four families (species overview in Figure 1). Glomeruli of all examined tadpoles showed anatomical segregation into distinct glomerular clusters. The conserved olfactory bulb architecture between the two members of early-diverging pipid frogs and later diverging neobatrachian frogs suggests an evolutionary constraint in the glomerular configuration in anuran tadpoles. Furthermore, we provide an outlook, that the organization of glomerular clusters in postmetamorphotic frogs might be more variable than in tadpoles.

## Materials and Methods

### Experimental Animals

Wild type and albino tadpoles of *Xenopus laevis* and larvae of wild type *Xenopus tropicalis* were bred and reared at the Institute of Animal Physiology, Justus-Liebig-University Giessen and kept in water tanks at a water temperature of 20°C and 25°C respectively. Tadpoles were fed with algae until the end of metamorphosis. *Ranitomeya variabilis* larvae and larvae and juveniles of *Dendrobates tinctorius* were bred and reared in the Department of Biology at Stanford University, Palo Alto, CA, USA. Individual tadpoles were kept separately after hatching at a water temperature of 25°C and fed with brine shrimp flakes and tadpole pellets, juveniles were kept in terraria and fed with flies. *Rhinella arenarum* tadpoles were obtained by *in vitro* fertilization from a colony at the Faculdad de Ciencias Exactas y Naturales of the University of Buenos Aires. Larvae of *Scinax granulatus* were collected from temporary ponds in the surroundings of the Campus of the University of Buenos Aires. All larvae were kept in tanks of dechlorinated water at 22°C and fed with chard leaves. The sex of the developing gonad in the tadpoles and juveniles was not determined in any of the species.

**Figure 2:**
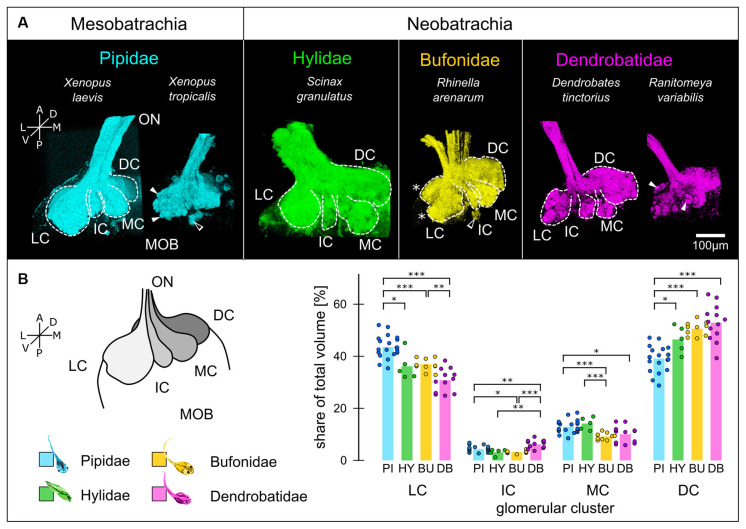
Glomerular clustering in the MOB is conserved among anuran tadpoles. **(A)** Glomeruli in the MOB of all species can be segregated into three ventrally (LC, IC, MC) and one dorso-medially located clusters (DC). White dotted lines—cluster outlines, filled arrowheads—glomeruli, empty arrowhead—small glomerular cluster, asterisks—ventral and dorsal lobes of the LC. **(B)** The relative volume of the clusters (schematically shown on the left) varies between the four families. The volumes of the clusters relative to the total glomerular volume for each family are shown. Each dot represents one MOB hemisphere and species of the same family are grouped. Significance levels: ***p < 0.001, **p < 0.01, *p < 0.05. A, anterior; P, posterior; L, lateral; M, medial; D, dorsal; V, ventral; ON, olfactory nerve; MOB, main olfactory bulb; LC, lateral cluster; IC, intermediate cluster; MC, medial cluster; DC, dorsomedial cluster; PI, Pipidae; HY, Hylidae; BU, Bufonidae; DB, Dendrobatidae.

An overview of the quantitatively analyzed samples of premetamorphotic larvae (Figure 2) is shown in Table 1. Experiments in higher staged tadpoles and juveniles were conducted for *Dendrobates tinctorius* (one animal stage 41, and two juveniles stage 45 after; Gosner, 1960) and *Xenopus tropicalis* (seven metamorphotic animals staged 55, 56, 58, 58, 61, 63 and 65 after Nieuwkoop and Faber, 1994).

**Table 1:** Overview of premetamorphotic animals examined in this study.

Species	Animals	Olfactory bulb hemispheres	Stages
*Xenopus laevis*	6	11	49–54 NF
*Xenopus tropicalis*	5	9	51–52 NF
*Scinax granulatus*	3	5	31–33 G
*Rhinella arenarum*	6	10	29–34 G
*Ranitomeya variabilis*	2	4	27 G
*Dendrobates tinctorius*	5	9	25–27 G

*Stages were determined according to Nieuwkoop and Faber (1994; NF) for the genus *Xenopus* and using the Gosner (1960) table (G) for all other species*.

### Tracing of Olfactory Projections

Animals were anesthetized in 0.02% MS-222 (ethyl 3-aminobenzoate methanesulfonate; Sigma–Aldrich, St. Louis, MO, USA) for approx. 5 min and placed on a wet paper towel under a stereoscope with fluorescent illumination. Olfactory projections from the nasal cavity to the MOB were labeled using Wheat-Germ agglutinin (WGA)-coupled fluorophores (10 μg/μl, WGA-Alexa Fluor 594/488, Thermo Fisher Scientific, Waltham, MA, USA) dissolved in frog Ringer (in mM: 98 NaCl, 2 KCl, 1 CaCl_2_, 2 MgCl_2_, 5 glucose, 5 Na-pyruvate, 10 Hepes, pH 7.8). The WGA-fluorophore solution (3–5 μl) was pipetted into the nostrils using a microloader and left to be taken up by ORNs for 10 min, while the animal was kept moist. Subsequently, the remaining solution was washed off and the animal transferred to a glass beaker to recover. After at least 24 h the animals were again anesthetized and killed by severing the spinal cord at the level of the brainstem. The olfactory nerves were transected close to the nose and the brain containing the olfactory nerves and the bulbs were dissected out of the tissue. The samples were either scanned immediately without fixation or fixed in 4% PFA in PBS for 1 h and imaged at a later timepoint.

### Imaging and Image Processing

The olfactory bulbs were imaged with the ventral surface up and fixed with nylon-stringed frames in a recording chamber. Image stacks were recorded at a z-resolution of 3 μm using multiphoton microscopy (upright Nikon A1R-MP and upright Leica SP5 multiphoton microscopes; excitation wavelength 780 nm) and processed in ImageJ (Schindelin et al., 2012). Since only one fluorophore was introduced per sample, we recorded pigmentation-derived auto-fluorescence by simultaneously recording with detectors of different emission-wavelength. In animal species with high pigmentation, we mathematically subtracted this auto-fluorescent signal from the images using the Image calculator function in ImageJ. Brightness and contrast were adjusted, and median filters were applied where necessary. Images showing both olfactory bulbs were stitched together using the stitching algorithm developed by Preibisch et al. (2009). All images presented are rendered in 3D using the 3D viewer plugin implemented in ImageJ.

### Volume Measurements and Statistics

Glomerular clusters were manually identified, contoured on various z-planes of the image stacks, and interpolated using the Segmentation Editor in ImageJ. The contours of a cluster were drawn according to the following criteria: clusters are spatially separated and connect to the olfactory nerve *via* axon fascicles. No fascicles between clusters were observed, thus resulting in a gap between two clusters. The segments labeled and measured did not include the nerve fibers projecting to these clusters. The volumes of the labeled clusters were analyzed using Python and their relative share of the total glomerular volume is presented in percentages. Averaged data are presented as mean ± standard deviation. For statistical analysis, the relative volumes of olfactory projections in each bulb hemisphere were considered as independent samples. Statistical significance was assessed using a One-Way ANOVA separately for each of the four clusters followed by Student’s *t*-tests for multiple comparisons. To control the familywise error, a Holm-Bonferroni correction was applied.

## Results

We first compared the larval glomerular organization by tracing the projections of ORNs from the MOE to the MOB (Figure 2). Wheat-Germ-Agglutinin (WGA) tracings of the most distal part of the left olfactory nerve (ON) and the glomeruli for each species are shown. Glomeruli can be discerned as spheroid accumulations of WGA (white arrowheads, Figure 2A). The glomerular array in all species is segregated into four unequally sized clusters (white dotted lines, Figure 2B), best described by their location as lateral (LC), intermediate (IC), medial (MC) and dorsomedial cluster (DC; Nezlin et al., 2003; Gaudin and Gascuel, 2005; Manzini et al., 2007). This organization is conserved between all species with only minor differences. The LC in *Rhinella arenarum* tadpoles show a clear bipartition into a dorsal and ventral lobe (white asterisk, bufonid tracings, Figure 2A). This bipartition is also present in the two *Xenopus* species, but not equally apparent as in *R. arenarum*. In addition to the four bigger clusters, a few ventro-posterior glomeruli could be observed in all species but were not clearly identifiable in all samples (empty arrowheads, Figure 2A; Brinkmann and Schild, 2016). Glomeruli in the DC were more clearly discernible in the Neobatrachians compared to *Xenopus*.

We then measured the percentual share of the clusters relative to the total glomerular volume (schematically shown in Figure 2B). Data from species belonging to the same family were pooled together. Across all species, the LC and the DC were most prominent with a combined relative volume of approx. 80%. In pipid tadpoles (*n* = 20 olfactory bulbs/11 animals; cyan in Figure 2B), the LC is slightly bigger than the DC, with 43.6 ± 4.3% and 39.1 ± 4.9% of the total volume, respectively. Contrastingly, in tadpoles of the other families (Hylidae: *n* = 5/3, green; Bufonidae: *n* = 10/6, yellow; Dendrobatidae: *n* = 13/7, magenta), the DC is bigger than the LC, with 46.5 ± 5.2% compared to 36.2 ± 5.4% in Hylidae, 50.6 ± 2.7% to 37 ± 2.1% in Bufonidae and 52.9 ± 6.9% to 30.8 ± 4.4% in Dendrobatidae. The percentual share of the LC in the Pipidae is significantly higher than in the other families (Figure 2B, left) and DC is significantly smaller (Figure 2B, right). The IC and the MC in all species are smaller than the other two clusters with approx. 5 and 10% of the total glomerular volume, respectively. The IC is biggest in the dendrobatid tadpoles (6.2 ± 1.6%) and significantly bigger than the IC in the other families (Pipidae: 4.4 ± 1.2%; Hylidae 3.2 ± 1.2, Bufonidae: 3.2 ± 0.5). The MC is smallest in the Bufonidae (9.3 ± 1.3%; Figure 2B) and biggest in hylid tadpoles (14.2 ± 2.4%). The MC of the dendrobatid tadpoles (10.1 ± 3.2%) is also smaller than in the pipids (12.9 ± 2.5%).

**Figure 3:**
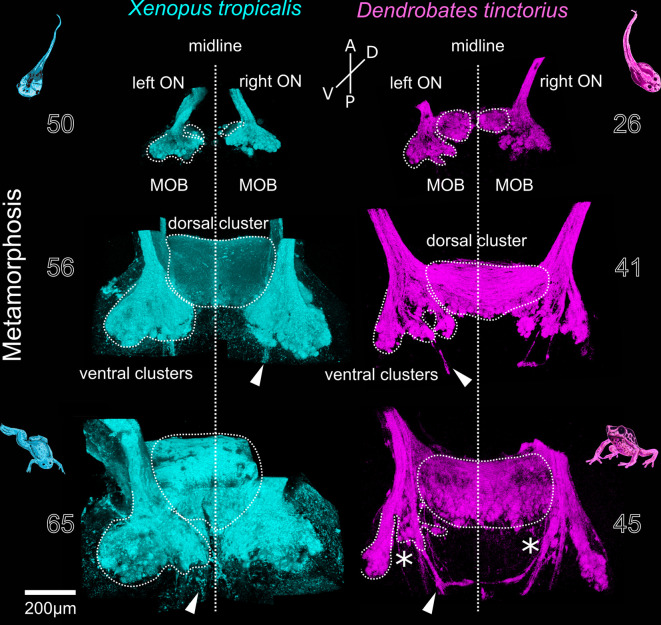
Metamorphotic changes of glomerular clusters in *Xenopus tropicalis* and *Dendrobates tinctorius*. Before metamorphosis (top), the left and right glomerular projections in the MOB are separated at the midline (vertical line). During metamorphosis, the dorsomedial components form an unpaired dorsal cluster (middle). In the late phases of metamorphosis (bottom), the ventral glomerular clusters in *X. tropicalis* are unchanged, while they are reduced in *D. tinctorius* (asterisks). Arrowheads—extrabulbar fibers. The numbers next to the images indicate the developmental stages after Nieuwkoop and Faber for *X. tropicalis* and Gosner for *D.tinctorius*. A, anterior; P, posterior; D, dorsal; V, ventral; ON, olfactory nerve; MOB, main olfactory bulb.

In most anurans, metamorphosis is accompanied by major habitat changes, which also impacts the olfactory system. We labeled glomeruli of the pipid *Xenopus tropicalis* and the dendrobatid *Dendrobates tinctorius* in different larval stages during metamorphosis and in early postmetamorphotic animals (Figure 3). In premetamorphotic tadpoles, the glomerular clusters of the left and the right MOB are separated at the interhemispheric midline (top images, Figure 3). At that stage, tadpoles of *D. tinctorius* already have a prominent dorsal glomerular region that extends towards the midline, while this region is less visible in *X. tropicalis* tadpoles. During metamorphosis, the dorsal glomerular regions of both sides start to medially fuse in both examined species (middle images, Figure 3). The ventral clusters (LC; IC; MC) are still present and remain unchanged. In both species, a bundle of axons bypasses the glomerular clusters, terminates in higher brain centers, or crosses the midline more caudally (white arrows, Figure 3). After the completion of metamorphosis (bottom images, Figure 3), the ventral clusters in *X. tropicalis* are unchanged, while they are reduced in *D. tinctorius* (asterisks, Figure 3). The LC is still clearly discernable in both species, while especially the IC and MC of *D. tinctorius* are not clearly delineated anymore.

## Discussion

During vertebrate evolution, a trend towards segregation into olfactory subsystems is apparent. Mammals possess several anatomically and molecularly distinct olfactory organs that project to different glomerular regions of the olfactory bulb (Munger et al., 2009; Bear et al., 2016). In fishes, ORNs expressing different odorant receptors are intermingled in a single olfactory surface (Hamdani and Døving, 2007). ORNs expressing receptors belonging to the same gene family often project to segregated glomerular clusters or regions in the olfactory bulb, putatively constituting functionally distinct odorant processing streams (Baier and Korsching, 1994; Hamdani et al., 2001; Frontini et al., 2003; Hansen et al., 2003, 2004; Sato, 2005; Braubach et al., 2012; Green et al., 2017). In anurans, a detailed analysis of the glomerular array is only available from the African Clawed frog *Xenopus laevis* (Gaudin and Gascuel, 2005; Manzini et al., 2007). Here we show that the organization of glomerular clusters is remarkably conserved between six distantly related anuran species despite a quite diverse eco-morphology of the examined tadpoles.

In larval *X. laevis*, glomerular clusters have been associated with a lateral and medial odorant processing stream (Manzini et al., 2002; Gliem et al., 2013). The medially located glomeruli in the MOB of *X. laevis* tadpoles are putatively innervated by ciliated ORNs expressing OR-type odorant receptors linked to G_α/olf_ and using the cAMP transduction pathway. The medial stream shows responses to alcohols, aldehydes, and ketones (Gliem et al., 2013). The lateral cluster on the other hand is highly responsive to amino acid stimulation and expresses G_α/o_and G_α/i_ linked to a cAMP-independent transduction pathway (Manzini and Schild, 2003; Gliem et al., 2013). Vomeronasal-type receptors (V1Rs or V2Rs), as well as trace-amine-associated receptors (TAARs), have been proposed to mediate this lateral stream (Date-Ito et al., 2008; Gliem et al., 2013; Syed et al., 2013). Even though these pathways are described quite in detail, their functional and behavioral significance is so far unknown. Amino acids are generally known as a potent food stimulus in fishes (Hamdani and Døving, 2007) and a lateral processing stream linked to feeding behavior has been identified in the olfactory bulb of the carp (Hamdani et al., 2001). Whether the lateral glomerular cluster in tadpoles also mediates feeding or foraging behavior remains to be elucidated (Terni et al., 2018).

Tadpoles of the examined species vary extensively in their feeding behavior, as they have adapted to a variety of microhabitats by differentiating into several morphotypes (Orton, 1953; Roelants et al., 2011). Earlier diverging frogs, like most pipids, rely on a large buccopharyngeal space to pump water through their body (Orton’s morphotype I), trapping mostly algae or other small food particles (Seale, 1982). However, derived phenotypes are more specialized, where the development of keratinized mouthparts enables tadpoles of Orton’s morphotype IV to rasp food particles off the ground or prey animals (Altig and Johnston, 1989; Roelants et al., 2011). For example, tadpoles of many dendrobatid frogs like *Ranitomeya variabilis* inhabit small temporary pools in bromeliads or leave axils, which often is linked to a scarcity of food resources (Altig and McDiarmid, 1999; Brown et al., 2008) and facultative cannibalism (Masche et al., 2010). In addition to their feeding behavior, there are major differences in social interactions. While tadpoles of *Xenopus* or *Rhinella arenarum* tadpoles are often seen forming schools for protection against predators (Altig and McDiarmid, 1999; Wells, 2007), *Dendrobates tinctorius* tadpoles engage in aggressive behavior against conspecifics (Fischer et al., 2020) and are transported by their parents if the food resources in the pool are exhausted (Altig and McDiarmid, 1999; Brown et al., 2008; Roland and O’Connell, 2015). While tadpoles rely on olfaction for foraging and kin recognition (Waldman, 1991; Veeranagoudar et al., 2004; Villinger and Waldman, 2005), it is currently unclear whether the olfactory system shows adaptation to specific ecological niches.

*R. arenarum*, like other anuran larvae, also has a part of their MOE exposed to the buccal cavity (Jermakowicz et al., 2004; Benzekri and Reiss, 2012; Jungblut et al., 2017). This buccal exposed epithelium is hypothesized to be important to assess food quality in species that actively scrape food off the substrate and is absent in suspension feeders like *X. laevis* (Jungblut et al., 2017). The glomerular projection target of this buccal exposed epithelium is currently unknown. It takes up approx. 15–20% of the entire epithelial volume in *R. arenarum* tadpoles (Jungblut et al., 2017) and is present in *Scinax granulatus* like in other hylid tadpoles (Jungblut et al., 2017; Jungblut personal observation). The presence of the buccal exposed epithelium in tadpoles of the two dendrobatid species tadpoles to our knowledge. Despite the eco-morphological difference and differences in the structure of the peripheral olfactory organ of the examined species, the spatial organization of glomeruli in the MOB of all examined tadpoles was remarkably similar. It is however still unclear, whether the anatomically similar clusters are also functionally or molecularly equivalent. In larval *R. arenarum*, both G_α/o_and G_α/olf_ are expressed in MOB glomeruli, but no clear segregation could be observed, in contrast to *X. laevis* tadpoles (Jungblut et al., 2009). It seems possible that the glomerular clusters are anatomically conserved but innervated by ORNs expressing receptors belonging to different receptor families, thus detecting different odorant cues. Whether the small variation in the relative volume of the described glomerular clusters e.g., the relatively bigger DC in the neobatrachians could be attributed to the presence of the buccal exposed epithelium or other ecological differences needs further experimental evidence.

After metamorphosis, the anatomy of the nose of different frog species is more variable than at the larval level (Helling, 1938). The larval MOE in the principal nasal cavity consisting of microvillous and ciliated ORNs transforms into the adult “air-nose,” solely consisting of ciliated olfactory ORNs (Föske, 1934; Bloom, 1954; Hansen et al., 1998; Reiss and Eisthen, 2008). In pipid frogs, a sensory epithelium consisting of both microvillous and ciliated ORNs forms in the middle cavity (Hansen et al., 1998) and seems to be a molecular and functional copy of the larval MOE (Hansen et al., 1998; Syed et al., 2017). In other anurans, a small patch of “aquatic” epithelium forms at the anterior bottom of the principal cavity, the recessus olfactorius, a putative homolog to the pipid middle cavity (Helling, 1938; Nowack et al., 2013). The middle cavity in these species is non-sensory (Reiss and Eisthen, 2008).

In the adult *Xenopus laevis*, ORNs located in the *de novo* formed middle cavity epithelium project to the ventrally located glomerular clusters, while the remodeled principal cavity connects to the dorsomedial glomeruli in the MOB (Reiss and Burd, 1997; Gaudin and Gascuel, 2005). The ventrally located glomerular clusters have been shown to remain anatomically (Gaudin and Gascuel, 2005) and functionally (Weiss et al., 2020) stable during metamorphosis, constituting a channel for waterborne olfaction. The dorsomedial region grows extensively and fuses at the midline, forming a single dorsal MOB (Gaudin and Gascuel, 2005). We show that the development of the dorsomedial portion of the MOB is similar between *Xenopus tropicalis* and *Dendrobates tinctorius* (Figure 3). Contrastingly, the ventrally located glomerular clusters are reduced in *D. tinctorius* after metamorphosis. Since *D. tinctorius* juveniles and adults are terrestrial, it is not clear to what extent they still rely on aquatic olfaction, in contrast to the fully aquatic *Xenopus*. Comparative data from different terrestrial and aquatic adults are needed to fully understand adaptive traits in the glomerular organization.

In conclusion, we show that the organization of glomerular clusters in eco-morphologically diverse tadpoles of distantly related species is remarkably conserved. It remains to be shown if the segregation into glomerular clusters also represents functionally distinct subsystems and if the small inter-species variability reflects possible adaptation to specific microhabitats.

## Data Availability Statement

The raw data supporting the conclusions of this article will be made available by the authors, without undue reservation.

## Ethics Statement

All experiments followed the guidelines of Laboratory animal research of the Ethics Committee of the University of Buenos Aires (CD: 316/12, Protocol #22), Justus-Liebig-University Gießen (GI 15/7 Nr. G 2/2019, 932_GP), and Stanford University (APLAC-33016).

## Author Contributions

LW, TH, and IM: conceptualization. LW: investigation, formal analysis, visualization, and writing. LW, LJ, AP, LO’C, TH, and IM: review and editing. LJ, AP, LO’C, TH, and IM: funding acquisition and resources. TH and IM: supervision.

## Conflict of Interest

The authors declare that the research was conducted in the absence of any commercial or financial relationships that could be construed as a potential conflict of interest.
